# Prevalence of smartphone addiction and its effects on subhealth and insomnia: a cross-sectional study among medical students

**DOI:** 10.1186/s12888-022-03956-6

**Published:** 2022-04-29

**Authors:** Huan Liu, Zhiqing Zhou, Long Huang, Ergang Zhu, Liang Yu, Ming Zhang

**Affiliations:** 1 Department of Hemodialysis, Yijishan Hospital of Wannan Medical College, Wuhu, Anhui 241001 China; 2 Department of Nursing, Yijishan Hospital Affiliated to Wannan Medical College, Wuhu, Anhui 241001 China; 3 School of Humanities and Management, Wannan Medical College, Wuhu, 241002 Anhui China; 4 School of Comprehensive Foundation, Wannan Medical College, Wuhu, 241002 Anhui China; 5 School of Innovation and Entrepreneurship, Wannan Medical College, Wuhu, 241002 Anhui China

**Keywords:** Prevalence, Smartphone addiction, Subhealth, Insomnia

## Abstract

**Objective:**

This study aimed to assess Chinese medical students’ smartphone addiction and its effects on subhealth and insomnia.

**Methods:**

A cross-sectional survey was conducted from October 14, 2020 to November 14, 2020 by administering an online questionnaire to Wannan Medical College students.

**Results:**

Of 2741 students who completed the survey, 1,447 (52.8%) had smartphone addiction. Medical specialty (*p* = 0.004), alcohol consumption (*p* = 0.001), smartphone use in bed (*p* = 0.000), depression (*p* = 0.000) and anxiety (*p* = 0.000) were strongly associated with smartphone addiction. The impacts of smartphone addiction on subhealth (*p* = 0.000) and insomnia (*p* = 0.000) were significant.

**Conclusion:**

This survey shows that the smartphone addiction detection rate among medical students was 52.8%. Students who did not like their medical specialty, consumed alcohol, used their smartphones in bed, and suffered from depression and anxiety had a higher smartphone addiction detection rate. The subhealth and insomnia of medical students are adversely associated with smartphone addiction.

**Supplementary Information:**

The online version contains supplementary material available at 10.1186/s12888-022-03956-6.

## Introduction

In line with the developing economy, the number of people using smartphones has been growing tremendously in recent years, and smartphones have become an organic part of every day. Smartphones are powerful devices that include many features, such as phone and internet browsers and access to social networks. According to a recent report, the number of smartphone users in 2021 was 3.8 billion [1]. Moreover, today’s college students are growing up with the company of smartphones, which have become a necessity in their lives [2]. Due to the convenience of smartphones, the abuse of smartphones increased significantly during the COVID-19 pandemic. As the number of smartphone users increases, smartphone addiction has also been rising. Several studies have reported that smartphone overuse can cause physical health problems for individuals, such as musculoskeletal pain, blurred vision, headache and pain in the wrists or neck [3]. Moreover, excessive use of smartphones is associated with numerous negative outcomes, including poor academic performance, academic procrastination [4], depression and anxiety, and poor sleep quality [5]. Researchers found that depressive and anxiety symptoms were highly prevalent among university students after the COVID-19 outbreak [6].

Although smartphone addiction is a common public health problem with a high prevalence reported in previous studies, there is a severe lack of epidemiological data about smartphone addiction among medical students during the COVID-19 pandemic in China. Smartphone use in China is very common among medical students [7]. The medical profession is one of the most stressful areas of university education because of its strong professionalism and high academic requirements. Subhealth among medical students is also an issue of academic interest. Previous studies have already reported a relationship between smartphone addiction and sleep quality [8]. Overall, evidence of subhealth and insomnia and their possible relationships with smartphone addiction among Chinese medical students is completely lacking.

Subhealth and sleep quality are severely affected in medical students due to high academic and clinical pressure. Using smartphones wisely plays a vital role in personal health. Although many studies on smartphone addiction have been conducted in China, to date, a literature search showed that no recent studies have investigated the relationship between smartphone addiction, subhealth and insomnia. Therefore, the purpose of this study was to determine the prevalence of smartphone addiction and to clarify the association between smartphone addiction and subhealth among medical students in a College of Medicine in China.

## Materials and methods

### Participants and procedure

This cross-sectional study web-based survey was conducted with medical students at Wannan Medical College between October 14, 2020 and November 14, 2020. The participants were undergraduates majoring in medicine from the freshman to senior year of Wannan Medical College. We invited students to participate using the widely popular Chinese social networking "QQ" and "WeChat" with the online survey available through the Wenjuanxing platform (https://www.wjx.cn/app/survey.aspx). Counselors’ and student leaders’ were recruited through the personal connections of the corresponding author of this article. These professional counselors and student leaders assisted in recruiting participants to complete this survey. According to the principles of the Declaration of Helsinki, the participants were informed of the purpose and significance of this study. The participants scanned the questionnaire QR code and completed the questionnaire anonymously in their own time. It took approximately 10 to 15 min for participants to complete the questionnaire.

### Measures

#### General demographic characteristics

Participants’ sociodemographic data, including gender, age, school year, place of residence, daily smartphone use time, etc. were collected.

#### Smartphone Addiction Scale (SAS)

The smartphone addiction test is a validated instrument to measure smartphone addiction. It contains 10 items that range from 1 (strongly disagree) to 6 (strongly agree), and cutoff scores are set at 31/60 for men and 33/60 for women to indicate the presence of smartphone addiction [9]. Cronbach’s alpha coefficient of the smartphone addiction test in this study was 0.81.

#### SHS evaluation

The condition of SHS was measured by using the self-reporting questionnaire SHSQ-25 [10], which is a widely used 25-item self-report questionnaire that evaluates subjective subhealth status in the previous three months. The 25 items yield seven component scores: fatigue, cardiovascular health, digestive tract, immune system, and mental health. For each item, there are five response categories (1 = never or almost never, 2 = occasionally, 3 = often, 4 = very often, and 5 = always). In the data analysis, never or almost never was 0, occasionally was 1, often was 2, very often was 3 and always was set to 4. The sum of scores for these seven components yields the SHSQ-25 total score, which ranges from 0 to 100. SHSQ-25 summed scores ≥ 35 were defined as “suboptimal health”, as has been previously demonstrated in the Chinese population. The Cronbach’s alpha coefficient of this scale in this survey was 0.834.

#### Insomnia Scale

Insomnia symptoms were measured using the Athens Insomnia Scale (AIS), which consists of eight items. Each item of the scale was scored on a four-point Likert scale from 0 (no problem) to 3 (very severe problem). The sum of the AIS scores is between 0 and 24, and the cutoff score of insomnia is 6 [11]. The AIS has been widely used and shows good reliability and validity in the Chinese college student population. In the present survey, the Cronbach’s alpha for the AIS was 0.90.

### Statistical analyses

The relationship between the demographic factors and smartphone addiction was analyzed using the chi-square test. Logistic regression was used to explore the influencing factors of smartphone addiction. All *P value*s were two-tailed, considering a *P* < 0.05 as statistically significantly associated with smartphone addiction.

### Ethics

This study design and procedure were approved by the ethics committee of Wannan Medical College. All participants provided their electronic informed consent for inclusion before participating in the study. These participants can withdraw at any time without providing any reason.

## Results

### Demographic characteristics

Among the 2741 students, a total of 1447 (52.8%) had smartphone addiction. Among the sample of 2741 medical students, 955 (34.8%) were male, and 1786 (65.2%) were female. Among them, 121 (65.1%) were dissatisfied with school life, 92 (66.2%) did not like to study their medical specialty, 65 (65.7%) smoked, 153 (65.9%) consumed alcohol, 1099 (57.5%) brought their phone to bed and 521 (36.4%) with self-perceived smartphone addiction had smartphone addiction demographic characteristics. The demographic characteristics of the study participants are presented in Table 1.

**Table 1:** Sociodemographic characteristics of the study sample (*N* = 2741)

Variable	Category	Overall n (%)	Smartphone addiction n (%)		*P* value
			**No**	**Yes**	
Gender	Male	955 (34.8)	472 (49.4)	483 (50.6)	0.089
	Female	1786 (65.2)	822 (46.0)	964 (54.0)	
Age	≤ 20	1647(60)	788 (47.8)	859 (52.2)	0.413
	≥ 21	1094(40)	506 (46.3)	588(53.7)	
School year	1st year	487(17.8)	260 (53.4)	227 (46.6)	0.022
	2nd year	786(28.7)	368 (46.8)	418 (53.2)	
	3rd year	646(23.6)	297 (46)	349 (54)	
	4th year	822(30)	369 (44.9)	453 (55.1)	
Place of residence	Rural	1759(64.2)	816 (46.4)	943 (53.6)	0.404
	Town	574(20.9)	274 (47.7)	300 (52.3)	
	City	408(14.9)	204 (50.0)	204 (50.1)	
Want to get a scholarship	Yes	2516(91.8)	1192 (47.4)	1324 (52.6)	0.556
	No	225(8.2)	102 (45.3)	123 (54.7)	
Student leader	Yes	809(29.5)	395 (48.8)	414 (51.2)	0.273
	No	1932(70.5)	899 (46.5)	1033 (53.5)	
Only child	Yes	916(33.4)	462 (50.4)	454 (49.6)	0.016
	No	1825(66.6)	832 (45.6)	993 (54.4)	
In love	Yes	682(24.9)	325 (47.7)	357 (52.3)	0.788
	No	2059(75.1)	969 (47.1)	1090 (52.9)	
School satisfaction	No	186 (6.8)	65 (34.9)	121(65.1)	0.000
	General	1263(46.1)	546(43.2)	717 (56.8)	
	Yes	1292(47.1)	683(52.9)	609(47.1)	
Do you like to study the medical specialty	No	139(5.1)	47(33.8)	92(66.2)	0.000
	General	1081 (39.4)	448(41.4)	633(58.6)	
	Yes	1521 (55.5)	799(52.5)	722(47.5)	
Smoking	Yes	99 (3.6)	34(34.3)	65(65.7)	0.009
	No	2642 (96.4)	1260(47.7)	1382(52.3)	
Alcohol consumption	Yes	232 (8.5)	79(34.1)	153(65.9)	0.000
	No	2509 (91.5)	1215(48.4)	1294(51.6)	
Bring your phone to bed	Yes	1910 (69.7)	811(42.5)	1099(57.5)	0.000
	No	831 (30.3)	483(58.1)	348(41.9)	
Hours of daily smartphone usage	≤ 1 h	73 (2.6)	35 (47.9)	38 (52.1)	0.000
	2 h	153 (5.6)	101(66.0)	52(34.0)	
	3 h	463 (16.9)	264 (57.0)	199 (43.0)	
	4 h	555 (20.2)	294 (53.0)	261 (47.0)	
	5 h	568 (20.7)	280 (49.3)	288 (50.7)	
	≥ 6 h	929 (33.9)	320(34.40	609(65.6)	
Self-perceived smartphone addiction	Yes	753 (27.5)	161(21.4)	592(78.6)	0.000
	No	1433 (52.3)	912(63.6)	521(36.4)	
	Not sure	555 (20.2)	221(39.8)	334(60.2)	

^*^p < 0.05, ** *p* < 0.01

### Logistic regression analysis of the factors associated with smartphone addiction

Multivariate analysis demonstrated that smartphone addiction was significantly associated with do you like to study your medical specialty, alcohol consumption, bringing your phone to bed, depression and anxiety. The associated factors of being at risk of smartphone addiction were not wanting to study the medical specialty, alcohol consumption, bringing your phone to bed, depression and anxiety (Table 2).

**Table 2:** Binary Logistic regression analysis of factors influencing smartphone addiction (*n* = 2741)

Variables	β	S.E	Wald	*P*	OR	OR 95% CI
Do you like to study medical speciality			11.064	0.004		
No	reference					
General	-0.13	0.202	0.416	0.519	0.878	0.591–1.304
Yes	-0.389	0.199	3.813	0.051	0.678	0.459–1.001
Alcohol consumption(1)	0.508	0.155	10.764	0.001	1.661	1.227–2.250
Bring your phone to bed (1)	0.593	0.09	43.793	0	1.81	1.518–2.157
Depression (1)	0.842	0.114	54.728	0	2.321	1.857–2.901
Anxiety (1)	0.591	0.114	26.977	0	1.805	1.444–2.255
Constant	-0.826	0.211	15.325	0	0.438	

### Relationship between smartphone addiction and subhealth and insomnia

Among the sample of 2741 medical students, 904 subjects (33.0%) were found to have SHS, and 781 (28.5%) of the participants scored above the cutoff score for insomnia. As shown in Table 3, the subhealth score of students with smartphone addiction was higher (r^2^ = 0.365, *p* < 0.001), and their insomnia was also higher than those without smartphone addiction (r^2^ = 0.566, *p* < 0.001).

**Table 3:** Pearson’s correlation among Sub-health, Insomnia, and smartphone addiction

**variables**	Sub-health		Insomnia	
	**r**	***P***	**r**	***P***
smartphone addiction	0.365**	0.000	0.566**	0.000

**p < 0.01

## Discussion

### Key findings

To the best of our knowledge, this was the first study on the relationship between smartphone addiction and subhealth, sleep quality, anxiety, and depression among medical students. This study identified the prevalence of smartphone addiction, insomnia, and subhealth among medical students as 52.8%. This is higher than another study that found that 29.8% of medical students in China had smartphone addiction [12]. Previous studies have shown that smartphone addiction is widespread among medical college students, and smartphone addiction has become a public health problem in China. According to a meta-analysis, the average prevalence of smartphone addiction among Chinese college students was approximately 23% [13]. A study of Lebanese university students found that 49% of students had smartphone addiction [14].

Smartphones are a “double-edged” sword that makes our lives convenient. The lives of many people and students are increasingly influenced by new technologies and devices, including smartphones. Due to the COVID-19 pandemic, young people had associated adverse psychological and behavioral effects, mainly related to infection control measures, which caused them to spend more time at home and mainly use technological tools. According to data released by the United Nations Educational Scientific and Cultural Organization (UNESCO), the COVID-19 pandemic is affecting nearly 363 million students from kindergarten to university around the world [15]. In China, more than 220 million students took online study courses at home instead of attending traditional school teaching models due to the impact of the COVID-19 pandemic [16]. Smartphones provided students the opportunity to continue their schooling. Compared with older people, college students are usually psychologically immature and have worse self-regulatory ability. Therefore, they are more likely to overuse smartphones [17].

### The impact of smartphone addiction on subhealth

Subhealth is a low-quality status between health and disease in the aspects of the physical, psychological and emotional performance of the subjects [18]. The World Health Organization (WHO) defines subhealth without organic pathological changes but functional changes as “the third state”, such as “chronic fatigue syndrome” [19, 20]. Smartphone addiction is well known to be closely associated with subhealth. Studies have shown that long-term online, high-frequency online and internet addiction are important risk factors for SHS [21], which is consistent with our research. During the COVID-19 pandemic, smartphones could be useful for many students as their main tool for communication, learning, entertainment, and information seeking. However, excessive use of smartphones may cause subhealth problems, such as fatigue, indigestion, and psychopathological problems, such as depression and anxiety. The average time of children and adolescents using smartphones per day increased during the COVID-19 pandemic [22]. In fact, during the COVID-19 pandemic, students had more sleep disorders, ocular alterations, and musculoskeletal diseases than before. Pain in the neck, shoulders, wrists and fingers are musculoskeletal diseases related to smartphone overuse and are reported frequently [23]. Smartphone addiction may also result in diseases such as dry eye disease, burning sensations in the eyes, conjunctival infection, decreased vision and macular degeneration [24, 25].

### The impact of smartphone addiction on insomnia

Our research indicated that there was a strong association between smartphone addiction and insomnia (r = 0.566) (*P* = 0.001) (Table 3). Studies have shown that college students with smartphone addiction are more likely to suffer from poor sleep quality. In a longitudinal study, Chen et al. reported that insomnia and nocturnal awakening difficulties were predictors of smartphone addiction [26]. In addition, studies have shown that smartphone addiction is correlated with daytime sleepiness, reduced duration of night sleep [27], later bedtime [27, 28] and poor sleep quality. First, excessive smartphone use at bedtime might postpone or interfere with sleep processes. Second, overuse of smartphones usually results in increased psychological stress, which also has a negative impact on sleep and physical recovery. Third, the blue light emitted by the screen might affect melatonin levels and thus affect sleep and wakefulness. Finally, the electromagnetic fields emitted from smartphones might also be one of the reasons for poor sleep quality. Due to the COVID-19 pandemic, students’ sleep time and wake-up time were delayed during school closures. Increasing evidence has shown that smartphone addiction is closely related to anxiety, depression, stress, impulsivity, and poor sleep quality [29, 30].

### Differences in smartphone addiction

Based on interpersonal theory, individuals with high levels of smartphone addiction often neglect real-world social interactions, leading to less personal companionship and lower social support, which leads to more anxiety and depression. In this stage, college students are willing to share their inner world with others, resulting in them gaining social support. As shown in the current study, a high level of smartphone addiction was a positive indicator of anxiety and depression. Anxiety symptoms had a positive relationship with smartphone addiction [31]. The main characteristics of COVID-19 are universal susceptibility and strong infectivity. The number of confirmed and suspected patients increased rapidly in a short period of time, putting people under a high degree of physical and psychological stress. In addition, to prevent the spread of the virus, people stayed home with restrictions on outdoor activities, such as having dinner, traveling, and going to school, which aroused public fear and anxiety. In addition, the shortage of prevention materials [32, 33] at the beginning of the pandemic caused panic and anxiety among a lot of people.

Similarly, poor lifestyle behaviors such as drinking and taking smartphones to bed are the main influencing factors of smartphone addiction. Adam summed up that the evening circadian typology was a risk factor [34]. Studies have also shown that adolescents with smartphone addiction have poor health [35] and poor academic performance [36]. Some experts have found that the lifestyle changes brought about by the COVID-19 pandemic have allowed people to develop enhanced behaviors, such as video gaming, movie watching, using social media, and internet surfing [37, 38]. Hysing et al. [39] reported that students who go to bed between 10:00 and 11:00 PM had the best grade point averages, while those who go to bed after 11:00 PM had worse grade point averages.

### Limitations

This study has several limitations. First, all behavioral, sleep and smartphone data in this study were derived from self-report questionnaire results, which may inevitably have bias. Second, because all participants in this study were enrolled in the same school, our conclusions must be conservative. Therefore, we will conduct follow-up research in the future.

## Conclusions

Smartphone addiction among Chinese medical students is a cause for concern because it may have a negative impact on their study, mood and physical health. Smartphone addiction has a great impact on sleep quality and subhealth among medical students. The results of this study revealed that studying the medical specialty, alcohol consumption, using the phone in bed, depression and anxiety were related to smartphone addiction.

## Supplementary Information

**Additional file 1.**

## Data Availability

The datasets used and/or analyzed during the current study are available from the corresponding author (H. L or M. Z) on reasonable request.

## Abbreviations

SAS: Smartphone Addiction Scale
SHS: Subhealth Status
AIS: Athens Insomnia Scale
WHO: World Health Organization
